# Prostaglandin D_**2**_ in Inflammatory Arthritis and Its Relation with Synovial Fluid Dendritic Cells

**DOI:** 10.1155/2013/329494

**Published:** 2013-05-07

**Authors:** Mahin Moghaddami, Enzo Ranieri, Michael James, Janice Fletcher, Leslie G. Cleland

**Affiliations:** ^1^Arthritis Research Laboratory, Hanson Institute, SA Pathology, Adelaide, SA 5000, Australia; ^2^Genetics and Molecular Pathology, Women's & Children's Hospital Campus, North Adelaide, SA 5006, Australia; ^3^Rheumatology Unit, Royal Adelaide Hospital, North Terrace, Adelaide, SA 5000, Australia

## Abstract

Prostaglandin (PG)D_2_ has been shown to be an active agent in the resolution of experimentally induced inflammation. This study was undertaken to determine the presence of PGD_2_ in chronic joint effusions and to explore the potential contributions of dendritic cells (DC) and monocytes to the intra-articular synthesis of PGD_2_. Synovial fluid (SF) was obtained from patients with inflammatory arthritis and knee effusions. PGD_2_ and PGE_2_ were detected in SF by ultrahigh-performance tandem mass spectrometry. Cellular fractions in SF were separated by density-gradient centrifugation and flow cytometry. The expression of hematopoietic prostaglandin D-synthase (hPGDS) and PGE-synthase (PGES) mRNA was determined by RT-PCR. Both PGD_2_ and PGE_2_ were detected in blood and SF, with PGD_2_ being more abundant than PGE_2_ in SF. mRNA for hPGDS was more abundant in SF mDCs than SF monocytes (*P* < 0.01) or PB monocytes (*P* < 0.001). SF mDC expressed significantly more hPGDS than PGES. Expressions of PGD_2_ and hPGDS were inversely associated with serum C-reactive protein (*P* < 0.01) and erythrocyte sedimentation rate (*P* < 0.01). The findings suggest that synovial DCs may be an important source of hPGDS and that systemic disease activity may be influenced by actions of PGD_2_ in RA and other arthropathies.

## 1. Introduction

Cyclooxygenase (COX) metabolises arachidonic acid to prostaglandin H_2_ (PGH_2_) which is then converted to PGE_2_ and PGD_2_ via their respective synthases [1]. Synthesis of PGE_2_ is involved in inflammation, and mice deficient in PGE synthase had decreased pain responses, decreased delayed-type hypersensitivity, and suppression of collagen-induced arthritis [2]. Upstream suppression of PGE synthesis by inhibitors of COX-2 is thought to explain the analgesic effects of nonsteroidal anti-inflammatory drugs (NSAIDs), including the COX-2 inhibitors, on arthritis [3]. 

In contrast to proinflammatory PGE_2_, PGD_2_ is active in resolving inflammation [4]. NSAIDs inhibit synthesis of PGD_2_ and have been shown to delay resolution of experimentally induced inflammation through a mechanism that can be overridden by local administration of exogenous PGD_2_ [4, 5]. There are two PGD synthase isozymes. Lipocalin-type PGD synthase is primarily expressed in brain, heart, and adipose tissue, and hematopoietic PGD synthase (hPGDS) is mainly expressed in mast cells, macrophages, dendritic cells (DC), and Th_2_ lymphocytes [6]. hPGDS appears to be the PGD synthase most involved in resolution of inflammation, since animals that are genetically deficient in hPGDS show impaired resolution of inflammation, and animals transgenic for hPGDS have reduced inflammatory responses [7]. The biological actions for PGD_2_ are mainly mediated via the D prostanoid receptors DP_1_ and DP_2_ (CRTH2) (for reviews, see [8, 9]). 

We have recently shown that dietary fortification with vitamin D_3_ reduced the severity and duration of adoptively transferred polyarthritis in rats and that the effect was associated with reduced expression of PGE synthase and increased expression of hPGDS by DCs from synovium-rich hind paw tissue [10].

PGE_2_ has been found in rheumatoid synovial fluid (SF) [11, 12] with emphasis on its role in inflammation and the effects of NSAIDs. While the potentially anti-inflammatory PGD_2_ and its nonenzymatic metabolite 15-deoxy PGJ_2_ have been found in synovial fluid, this prostaglandin has received much less attention [13, 14]. We have examined chronic joint effusions for the presence of PGD_2_ and have explored the potential contributions of myeloid DC (mDC) and monocytes to the intra-articular synthesis of PGD_2_. 

## 2. Materials and Methods

### 2.1. Subjects

SF and blood samples were obtained from patients undergoing arthrocentesis of chronic inflammatory knee effusions. All patients were ambulatory community dwellers, and, with the exception of two patients, the remainder were known to be vitamin D replete based on serum 25-hydroxy-cholecalciferol being ≥74 nmol/L, or based on history of regular vitamin D supplementation. While the vitamin D status in the other two patients was not known, both were active in outdoor pursuits. Diagnostic details and medications taken are shown in Table 1. All patients and healthy donors gave informed consent and the study protocol was approved by the Human Research Ethics Committee, Royal Adelaide Hospital Australia. 

### 2.2. Measurement of Systemic Disease Activity

Systemic disease activity was assessed by routine laboratory testing for erythrocyte sedimentation rate (ESR) and C-reactive protein (CRP). 

### 2.3. PGD_2_ and PGE_2_ Assay

Analysis of PGD_2_ and PGE_2_ by ultrahigh-performance liquid chromatography tandem mass spectrometry (UPLC-MS/MS) was undertaken based on the protocol reported by Unterwurzacher et al. [15]. The method enables the resolution of PGE_2_ and PGD_2_ with quantification using homologous deuterated internal standards. Unfractionated SF and whole blood samples were applied to purpose-designed filter papers and stored at −70°C until further use. Analysis of 6 mm punched filter paper spots was extracted with 100 *μ*L of acetonitrile : water mixture containing the stable isotopes. A volume of 10 *μ*L was injected onto a reversed phase column (ZORBAX Eclipse XDB C18, 3.0 × 100 mm, 3.5 *μ*m particle size, Agilent Technologies, Vienna, Austria). The prostanoids were eluted using a gradient of water to 100% acetonitrile/0.05% formic acid into a AB Sciex API 5000 QJet triple quadrupole instrument. The concentration of each analyte was determined against the respective stable isotope prostaglandin D_2_-d4 (PGD_2_-d4) and prostaglandin E_2_-d4 (PGE_2_-d4) from Cayman Europe Chemicals. 

### 2.4. Isolation of Mononuclear Cells from SF and Peripheral Blood (PB)

SF and blood samples were collected into heparinised tubes. SF was diluted in RPMI containing 2% fetal bovine serum (complete medium) prior to centrifugation for 10 minutes at 293 g [16]. The SF cells were resuspended in complete medium. Resuspended SF cells and blood cells were fractionated by centrifugation on a Lymphoprep density gradient at 600 g for 30 minutes in order to isolate mononuclear cells. All cell analyses were undertaken on freshly isolated cells. 

### 2.5. Flow Cytometric Analysis

Flow cytometric analysis was performed as described previously [10]. The following antibodies were used: Alexa fluor 488 anti-human CD11b, HLADR PE-cy5, PE-CD11c, PE-CD163, PE-CD14, and APC-CD14 (BD Biosciences, San Jose, CA, USA). Relevant isotype controls were used throughout. Labelled cells were analysed with a Beckman Coulter EPICS XL-MCL flow cytometer and Coulter EXPO 32 software (Beckman Coulter, Fullerton, CA, USA). 

### 2.6. Isolation of Myeloid DCs and Monocytes by Flow Cytometry

SF mononuclear cells separated by gradient density were labelled with a cocktail of conjugated mAbs comprising Alexa fluor 488-CD11b, phycoerythrin CD11c, PE-cy5 HLADR, and APC-CD14 for 45 min at 4°C as described previously [10]. Cells were gated by size (Figure 2(a)) and subsequently sorted into CD11b+ HLADR+ CD11c+ CD14− (mDC) and CD11b+ HLADR+ CD11c+ CD14+ (monocytes) (Figure 2(e)) populations, using FACS Diva software (Becton Dickinson), as described previously [17]. PB blood mononuclear cells were labelled with PE anti-CD14 mAb followed by separation of CD14+ cells by cell sorter. 

### 2.7. Cytology

Cytospin smears prepared from flow cytometrically sorted cells were fixed and stained as described [18].

### 2.8. RNA Isolation and Quantitative RT-PCR Analysis of Gene Expression

Total RNA was extracted from flow cytometrically sorted cell populations using the RNeasy Mini Kit (Qiagen, Valencia, CA, USA) according to the manufacturer's instructions. Total RNA was reverse-transcribed to cDNA and amplified using the two-step reverse transcription-polymerase chain reaction (RT-PCR) kit from Qiagen. RNA and cDNA quality was assessed using a NanoDrop 1000 spectrophotometer (ThermomFisher Scientific, Wil ington, DE, USA) before samples were frozen at −70°C until further use. Gene expression levels were investigated using commercially available specific primers for human genes obtained from Qiagen including hPGDS (QT-00022043), PGES (QT-00208607), DP_1_ (QT-00036190), and DP_2_ (QT-00042448). Real-time PCR was performed using the QuantiFast SYBR Green PCR kit (Qiagen) according to the supplier's protocol, in a Rotor-Gene 3000 real-time PCR machine (Corbett Research, NSW, Australia). A minimum of three replicates of each sample was amplified in all experiments. Each PCR had a sample prepared without template and a sample prepared without primers, serving as negative controls. The reactions were incubated at 95°C for 5 min followed by 35 cycles of 95°C for 10 s and 60°C for 30 s. PCR product quality was monitored using post-PCR melt curve analysis. Fold changes were calculated using the formula 2^−(ΔCt)^, where ΔCt = Ct (target gene) − Ct (*β*-actin) [19]. 

### 2.9. Statistical Analysis

All data were analyzed using GraphPad Prism V5.0 (GraphPad Software, Inc., San Diego, CA, USA). Quantitative real-time RT-PCR signals were normalized to *β*-actin. One-way analysis of variance (ANOVA) with Newman-Keuls post hoc test was used to determine significant differences between groups. C-reactive protein (CRP) and ESR correlation to hPGDS expression by SF mDC was analysed by Pearson's correlation. *P* < 0.05 was considered statistically significant. 

## 3. Results

### 3.1. PGD_2_ and PGE_2_ in Inflammatory Synovial Fluid

**Figure 1:**
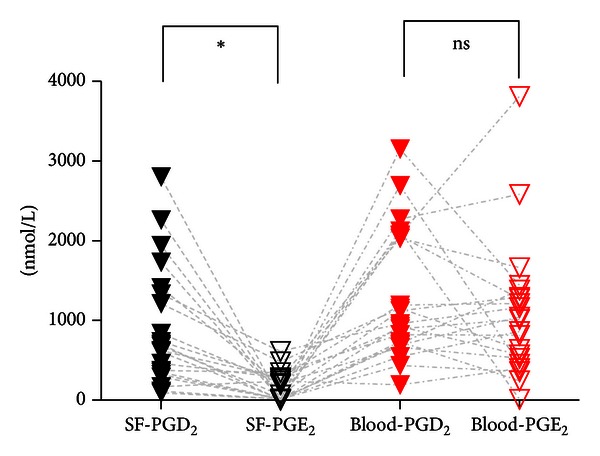
Concentrations of PGD_2_ and PGE_2_ in synovial fluid and whole blood. Lines connect results from the same person. *P < 0.01; ns: not significant.

There were similar concentrations of PGD_2_ and PGE_2_ in whole blood (Figure 1) whereas the concentration of PGD_2_ was substantially greater than that of PGE_2_ in knee effusions from patients with inflammatory arthropathies (*P* < 0.01) (Figure 1). 

### 3.2. Expression of hPGD and PGE Synthases by Myeloid Dendritic Cells (mDCs) and Monocytes Isolated from Synovial Fluid

**Figure 2:**
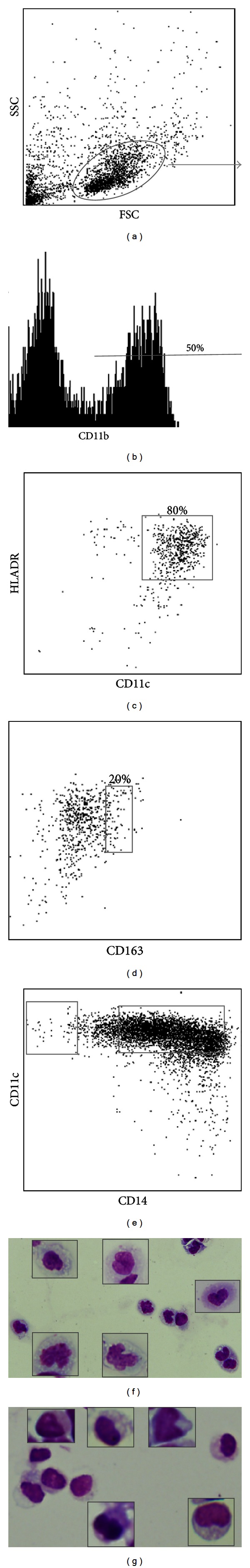
Flow cytometric analysis of cells prepared from SF aspirates. (a) Shows the selected gate based on forward and side scatter of light. (b) Shows CD11b staining of 50% of SF cells in a single peak. (c) Shows 3-colour flow cytometric analysis in which 80% of CD11b+ HLADR+ SF cells express CD11c. (d) Shows 3-colour analysis of SF cells in which 20% of CD11b+ HLADR+ cells express CD163. (e) Shows 4-colour analysis to separate CD14− from CD14+ subsets of HLADR+ CD11b+ CD11c+ cells. The CD14− cells were enriched for cells with DC morphology (f), whereas the CD14+ subpopulation displayed monocyte morphology (g).

Populations of mononuclear cells in SF were characterised by three- and four-colour flow cytometry. The forward light scatter gate was chosen (Figure 2(a)) to exclude most lymphocytes and neutrophils and to include monocytes, macrophages, and DCs. CD11b antibody, which detects myeloid haematopoietic cells, stained about half of the cells in this gate. The CD11b stained cells comprised a single peak of fluorescence, which was well resolved from unlabelled cells (Figure 2(b)). About 80% of the CD11b+ HLADR+ cells in SF expressed CD11c (Figure 2(c)), consistent with their designation as monocytes or mDC. The minority population (20%) of CD11b+ HLADR+ cells appeared to be macrophages as evinced by proportionate staining for the macrophage marker CD163+ (Figure 2(d)). Based on CD14 staining in four-colour flow cytometric (Figure 2(e)) and cytospin analyses (Figures 2(f) and 2(g)), the CD11b+ HLADR+ CD11c+ cells were separated into mDC (5%) (CD14−, Figure 2(e); DC morphology, Figure 2(f)) and monocytes (75%) (CD14+, Figure 2(e); monocyte morphology, Figure 2(g)). 

**Figure 3:**
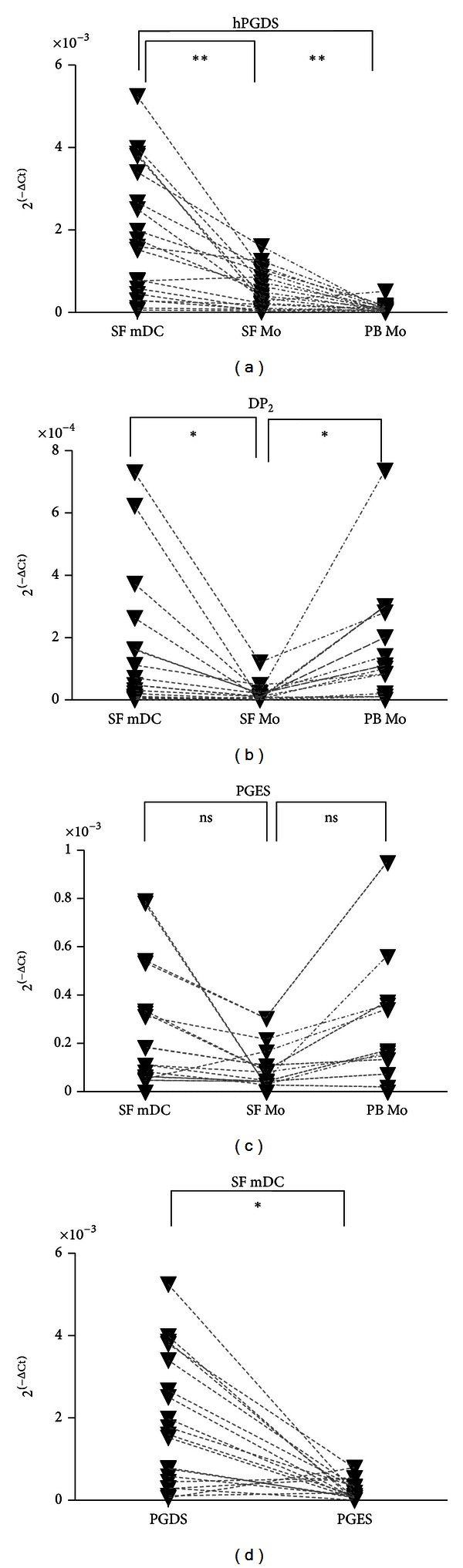
Expression of mRNA for (a) hPGDS, (b) PGD receptor DP_2_, (c) PGES in SF myeloid DC, SF monocytes, and PB monocytes, and (d) hPGDS versus PGES in SF mDC in patients with inflammatory arthritis. *P < 0.01, **P < 0.0001, ns: not significant.

The expression of mRNA for hPGDS by SF mDC was greater in SF mDC than in SF monocytes (*P* < 0.0001) or peripheral blood monocytes (*P* < 0.0001) (Figure 3(a)). mRNA for the PGD receptor DP_2_ was increased in SF mDC and peripheral blood monocytes compared with SF monocytes (Figure 3(b)). Minimal mRNA expression of enzymes COX-1 or COX-2 (data not shown) or the potentially competing terminal synthase PGES (Figure 3(c)) was detected in SF mDC and monocytes. As shown in Figure 3(d), in SF mDC expression of hPGDS was significantly (*P* < 0.05) higher than PGES. 

### 3.3. PGD_2_ Expression and Disease Activity

**Figure 4:**
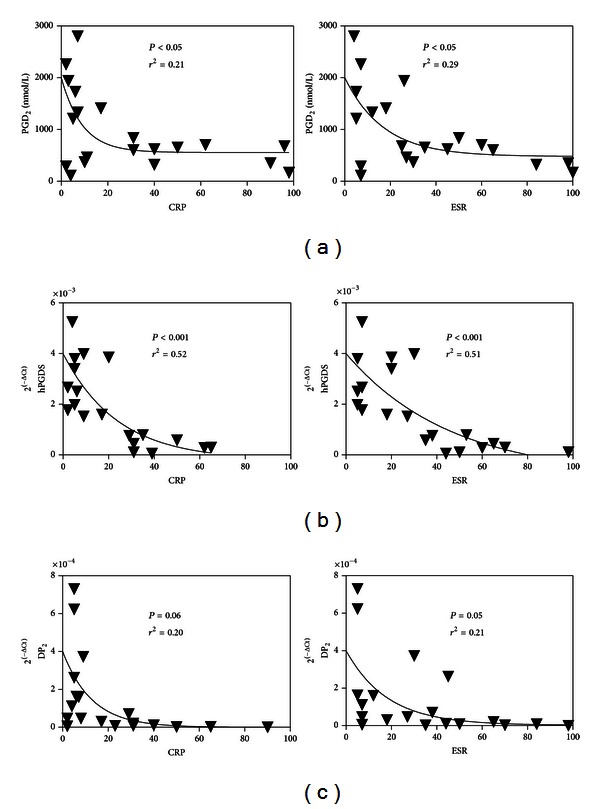
Pearson's correlations between systemic disease activity measured by CRP or ESR and (a) synovial concentrations of PGD_2_, and synovial mDC expression of (b) hPGDS and (c) DP_2_ receptor.

The higher level of PGD_2_ in synovial fluid and higher expression of hPGDS by SF mDC seen in some patients was intriguing and led us to explore a possible correlation with inflammatory disease activity (Figure 4). CRP and ESR levels were inversely correlated with synovial fluid concentration of PGD_2_ and synovial fluid mDC expression of hPGDS (Figure 4). 

## 4. Discussion

The progression from acute to chronic inflammation has been viewed as a persistence of excess proinflammatory mediators, but more recent studies show that it may also arise from a failure of mechanisms that resolve inflammation [20]. Although mononuclear cells can in many settings contribute to proinflammatory responses, they are also critical in tissue repair in a noninflammatory manner [21]. Most successful inflammatory processes are self-limiting, which implies the existence of endogenous anti-inflammation pathways [22]. 

A number of mediators, including PGD_2_, have been shown to actively promote resolution of inflammation [22, 23]. PGDS knockout mice had impaired resolution of inflammation whereas PGDS transgenic mice had reduced inflammatory responses [7]. PGD_2_ suppresses joint inflammation in murine collagen-induced arthritis [24]. We have shown that dietary fortification with vitamin D3 reduced the severity and duration of adoptively transferred polyarthritis (ATA) in rats, and this was associated with increased expression of hPGDS and reduced expression of PGES by mDC from synovium-rich hind paw tissue of arthritic rats [10]. The source of SF PGD_2_ probably involves cells other than SF mDCs. Mast cells contain PGDS and anti-IgE stimulates PGD_2_ synthesis in human and rat mast cells [25]. However, antigen presenting cells including DC were the major source of hPGDS in various rat tissues even though there was expression in mast cells [26]. In human skin, all of the antigen presenting cells express hPGDS, and this includes Langerhans cells, dermal DCs, and plasmacytoid and myelocytic DCs. The authors reporting these observations acknowledge that mast cells produce PGD_2_ but conclude that epidermal DCs such as Langerhans cells should be a major source of PGD_2_ in skin at least [27]. While there appear to be no prior reports linking SF mDCs to production of PGD_2_, monocyte-derived DCs have been shown to produce PGD_2_ [27]. 

In the present study, we observed that SF from patients with inflammatory arthritis contains significantly greater levels of PGD_2_ than PGE_2_. SF mDCs were found to express significantly more hPGDS than PGES. PGD_2_ may affect various immune cells and effector cells, including mDCs themselves through the DP_2_ receptor, which we found is more strongly expressed in SF mDCs and PB monocytes than in SF monocytes. Exogenous PGD_2_ and its nonenzymatic metabolite 15-deoxyΔ12,14 PGJ_2_ (15d-PGJ_2_) has the ability to modulate the function and maturation of monocyte-derived DC [28, 29]. While 15d-PGJ_2_ can interact with the DP_2_ receptor, there is longstanding controversy as to whether it is an endogenous mediator, especially of PPAR*γ*, due to its very low levels in vivo [30]. Factors which enhance the expression of PGD synthase should have useful anti-inflammatory effect. The participants in this study were replete in vitamin D which we have shown to upregulate joint mDC hPGDS in rat polyarthritis [10]. 

There were no significant differences in the expression of PGES between SF mDCs or SF or peripheral blood monocytes. Both SF mDCs and monocytes expressed little COX-2 constitutively, although we have observed that COX-2 expression is significantly upregulated by these cells in response to LPS stimulation (data not shown). As mentioned above, SF mDCs expressed significantly lower level of PGES than PGDS. It is possible that the anti-inflammatory treatments applied may have influenced PGES expression [31, 32]. Our practice is to advise patients to avoid NSAIDs in favour of an anti-inflammatory dose of fish oil and to use NSAIDs sparing as needed for 2nd-line analgesia on grounds of safety and the lack of a favourable disease modifying effect with NSAIDs use. On mechanistic grounds, one might expect regular, more intensive NSAIDs use to yield symptomatic benefit from reduced PGE_2_ synthesis achieved through the reduction of the precursor COX-2 product PGH_2_, which is the substrate for both PGES and PGDS. However, a concomitant reduction in PGD_2_ synthesis would be expected to compromise disease control and resolution. While conventional and biological disease modifying antirheumatic drugs (DMARDs) may conceivably influence the production of PG, the effects are likely indirect. The issue has been most thoroughly investigated with methotrexate with conflicting findings [33–35]. 

hPGDS and its products PGD_2_ and further downstream metabolites 15-deoxyΔ12,14 PGJ_2_ (15-PGJ_2_) are clearly involved in resolution of inflammation, acting on cell traffic and cytokine synthesis in animal models [5, 7, 36]. Colonic mucosal synthesis of PGD_2_, which is specifically upregulated during remission from ulcerative colitis, may contribute to the maintenance of remission in these patients [37]. The finding of hPGDS in human synovial mDC as well as PGD_2_ in synovial fluid and the inverse relation to disease activity prompts the question of whether mediators present in inflamed joints are inducers of hPGDS in human SF DCs. If so, suppression of the synthesis or action of these mediators by NSAIDs may suppress the development of the natural resolution phase of inflammation. 

In conclusion, the findings indicate that synovial mDCs exhibit expression characteristics appropriate for an active role in PGD_2_ synthesis and that PGD_2_ is present in inflammatory effusions. The inverse correlation of both PGD_2_ and expression of hPGDS in mDCs in SF with the markers of systemic disease activity (CRP and ESR) suggests that systemic disease activity may be influenced by actions of PGD_2_ in rheumatoid arthritis and other arthropathies. Within this small sample of patients, most of whom had rheumatoid arthritis, this putative effect was not obviously influenced by the type of arthropathy or DMARD therapy. It remains to be determined if the elevated PGDS in SF mDCs and PGD_2_ in SF observed in patients who had low CRP and ESR contributes to remission in RA group of patients. 

## Figures and Tables

**Table 1:** Demographic and clinical characteristics of patients.

Diagnosis	Age (years)	Sex (years)	Disease duration	Treatment
RA	83	F	18	DMARDs + NSAIDs + vitD + fish oil
RA	53	F	3	DMARDs + NSAIDs + fish oil
RA	82	F	30	DMARDs + vitD + fish oil
RA	48	F	22	DMARDs + NSAIDs + fish oil
RA	61	F	13	Tocilizumab + fish oil
RA	57	F	27	DMARDs + Adalimumab + fish oil
RA	60	F	38	DMARDs + vitD + fish oil
RA	62	F	2	NSAIDs
RA	82	F	11	DMARDs + fish oil
RA	50	F	1	DMARDs + vitD + fish oil
RA	66	F	10	DMARDs + vitD
RA	67	F	29	DMARDs + vitD
RNP^+^ polyarthritis	55	F	3	DMARDs + NSAIDs + vitD + fish oil
B27^+^ Pauci-arthritis	35	F	18	DMARDs + vitD + fish oil
Psoriatic arthritis	58	F	6	DMARDs + vitD + fish oil
Psoriatic arthritis	55	F	2	Fish oil
B27^+^ spondyloarthritis	62	M	41	DMARDs + NSAIDs + fish oil
Juvenile onset-monoarthritis	34	F	26	NSAIDs
Crohn's arthritis	46	F	30	DMARDs + vitD
Crohn's arthritis	70	F	13	DMARDs + NSAIDs + vitD + fish oil

RA: rheumatoid arthritis; DMARDs: disease-modifying anti-inflammatory drugs; NSAIDs: nonsteroidal anti-inflammatory drugs.
